# Ultra-fast green microwave assisted synthesis of NaFePO_4_-C nanocomposites for sodium ion batteries and supercapacitors

**DOI:** 10.1038/s41598-022-20329-x

**Published:** 2022-09-29

**Authors:** Wael Wazeer, Marwa M. Nabil, Mohamed Feteha, Moataz B. Soliman, Abd El-Hady B. Kashyout

**Affiliations:** 1 Electronic Materials Department, Advanced Technology and New Materials Research Institute, City of Scientific Research and Technological Applications (SRTA-City), P.O. Box 21934, New Borg El-Arab City, Alexandria Egypt; 2 Materials Science Department, Institute of Graduate Studies and Research, Alexandria University, 163 Horrya Avenue, Shatby, P.O. Box 832, Alexandria, 21526 Egypt

**Keywords:** Chemistry, Energy science and technology, Materials science, Nanoscience and technology

## Abstract

Sodium ion batteries are favored in stationary and large scale power storage due to their low cost and nontoxicity. As the lithium is replaced with sodium due to the cost motive, a cheap processing method is needed to maintain the cell price as low as possible. We report an ultra-fast synthesis method that utilizes the high microwave absorbance of silicon carbide content in rice straw ash. Amorphous/maricite mixtures of sodium iron phosphates-carbon composites (NaFePO_4_-C) are synthesized, crystallized, and carbon coated using one-step microwave heating. The sodium ion electroactive composites are prepared using different microwave heating durations ranging from 30 to 100 s. High purity inert gases are not needed during synthesis, processing, and even at cell assembly. The materials are characterized by elemental analysis techniques, X-ray diffraction (XRD), scanning/transmission electron microscope (SEM/TEM), and Raman spectroscopy. The electrochemical performance of the synthesized nanocomposites is examined as sodium ion battery cathode and as symmetric supercapacitors. The optimum synthesis time is 60 s for the application as sodium ion batteries and as a supercapacitor. The maximum specific capacity is 108.4 mA h g^−1^ at 0.2 C in the case of using it as a battery cathode. While the capacitance is 86 F g^−1^ at 0.5 A g^−1^ as a supercapacitor. The capacity retention is 92.85% after 40 cycles at 0.2 C as sodium ion battery electrode. For supercapacitor, the capacity retention is 81.7% after 1000 cycles.

## Introduction

Mobile applications such as electric vehicles (EVs), laptops and cell phones have been invaded by lithium ion technologies due to the high specific gravimetric capacities of Li cells^1^. The lithium depletion fears came early to put the basics of their replacement with sodium ion technologies, especially in stationary and large scale common applications^2,3^. Sodium has many characteristics that are very close to those of lithium, especially their electronegativity and ionic radius. On the other hand, sodium is environmentally friendly and its abundance in the earth's crust is about 1350 times more than that of lithium^4^. Many nanomaterials have been revealed as cathodes and anodes for sodium ion batteries (SIBs) and have been published elsewhere^5–8^. Sodium vanadium phosphates (NVP) Na_3_V_2_(PO_4_)_3_ and Na_3_VCr(PO_4_)_3_ with NASICON structures were among the most successful active cathode materials owing to their high and flat discharge potential at 3.4 V vs Na/Na^+^, which maximizes energy density. They also have the high rate capability advantage due to their sodium ion superconducting open framework structure. However, only two of the three Na^+^ ions are available at this high potential, resulting in a relatively low theoretical capacity of 118 mA h g^−1^. In addition, their cost is relatively high^9,10^. As a raw material, iron oxide is one tenth the cost of vanadium oxide, which lowers the cost advantage of using NVP when compared with LiFePO_4_^11^. The sodium iron phosphate with its different forms provides a cheap material as sodium ion battery cathodes, in addition to their environmental safety^5^. Maricite, Olivine, and amorphous forms of NaFePO_4_, all with a theoretical capacity of 152 mA h g^−1^, have been explored as active, low cost cathode materials for SIBs^12–16^. Olivine NaFePO_4_can be prepared only from LiFePO_4_ by electrochemical delithiation and subsequent sodiation. It possesses low ionic conductivity due to its one direction Na/Li diffusion tunnels^12,17^. The conversion of maricite NaFePO_4_ to amorphous phase was reported by Kim et al*.* after full de-sodiation, so that, maricite and amorphous NaFePO_4_ can be considered to have the same electrochemical impact as cathode materials^13^.

Alkali metal-free transition metal phosphates are attractive materials for symmetric and asymmetric supercapacitors and many reports have been reviewed by Li et al.^18^. In a limited number of articles, Li/Na transition metal phosphate materials that were originally active as Li/Na ion cathode in batteries have been utilized as an active materials in asymmetric electrochemical supercapacitors. They exhibited good capacitances and excellent cyclic stabilities^19–21^. NaNiPO_4_ delivered 125 F g^−1^ when 2 M of NaOH was used as electrolyte and the voltage range was 0–1.6 V^20^. Sundaram et al*.* prepared NaMn_1/3_Co_1/3_Ni_1/3_PO_4_ with Maricite structure, the asymmetric supercapacitor versus carbon that reported a specific capacitance of 40 F g^−1^ when 1 M NaPF_6_ in EC/DMC was used as electrolyte and the voltage range was 0–3 V^21^.

Microwave solid state methods provided a very fast and low-cost route for the synthesis of alkali-transition metal phosphates, especially for production of olivine LiFePO_4_^22–24^. But microwave methods were uncommon for synthesizing Na-transition metal phosphates^25–27^. In this work, sodium iron phosphate is examined as both sodium ion battery and supercapacitor for the first time. The precursor materials cost, the synthesis method cost and the time consumed were greatly minimized by the current procedures. Moreover, a cheaper current collector (Cu foil instead of Ni in the case of supercapacitors) and a dry air filled glove box are used (instead of argon filled glove box). The current work is aiming at lowering the overall battery production cost as much as possible, which is the main target of using sodium ion technologies.

## Experimental

### Materials synthesis

**Figure 1:**
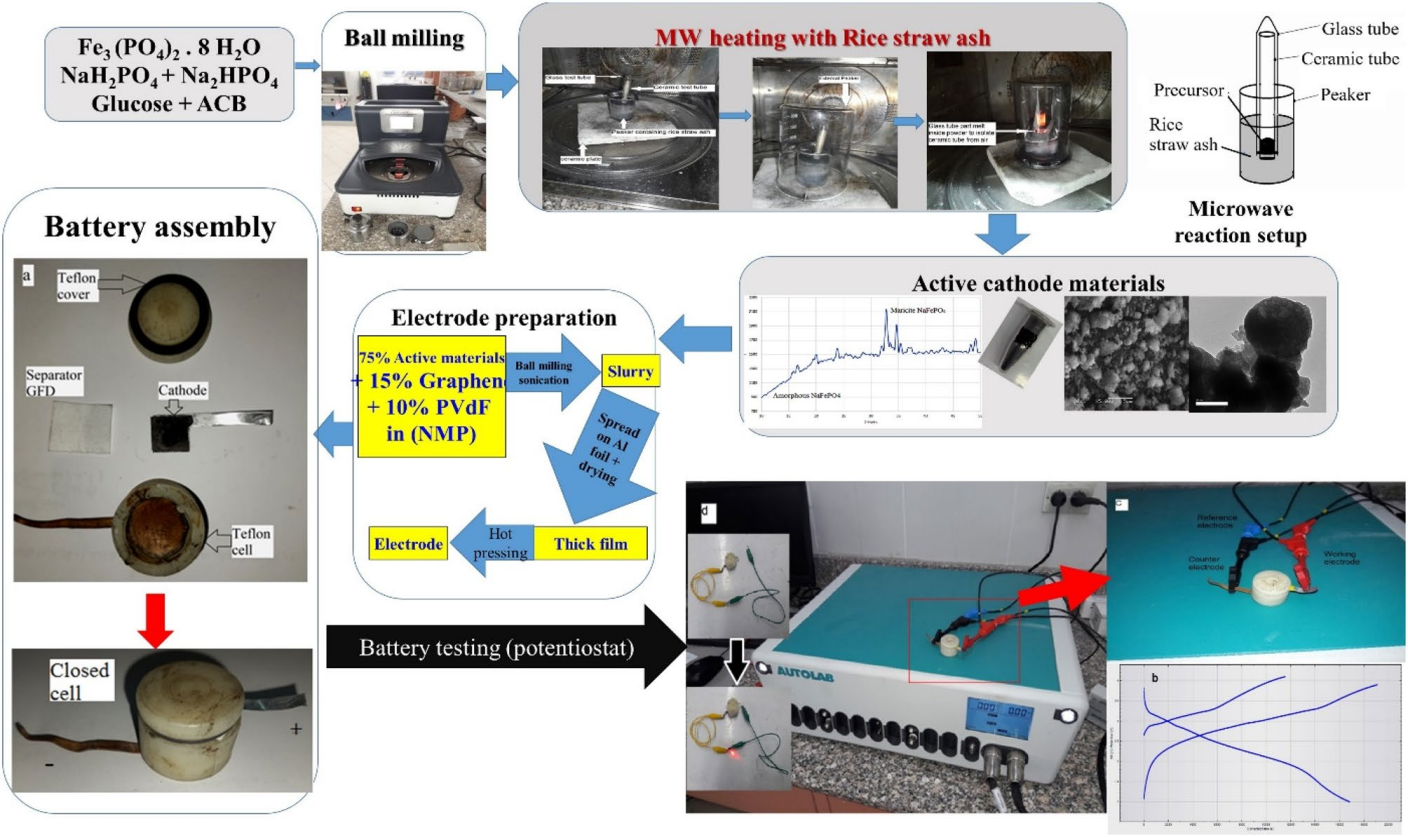
Synthesis process of sodium iron phosphate through ball milling and microwave assisted and assembly of related batteries and supercapacitors.

The active materials were synthesized using a microwave carbothermal solid state method. A mixture of 5.01 g Fe_3_(PO_4_)_2_·8H_2_O (prepared in the laboratory by procedures detailed in supporting information), 0.6 g NaH_2_PO_4_ and 2.86 g Na_2_HPO_4_ (99.5%, Polskie Odczynniki Chemiczne s.a. Belgium) were ball milled with 3 wt% (0.215 g) of commercial glucose (Anhydrous, ADWIC El-Nasr company, Egypt) and 5wt% (0.355 g) of activated carbon black (ACB) (99.99% VULCAN XC72R,CABOT, USA) at 500 rpm for 30 min in a 50 ml vessel using rotating mode ball milling machine (Fritsch Pulverisette 7, Germany). The ball to powder ratio was 10:1, and the stoichiometry of Na:Fe:P was 3:2:3, (initially designed for the synthesis of NASICON Na_3_Fe_2_(PO_4_)_3_). For microwave heating, 10 g of previously heat treated rice straw powder was placed in a glass beaker, while a ceramic tube containing 0.8 g of the precursor mixture was immersed in the black rice straw ash powder. The ceramic tube was covered by a 5 ml Wassermann test tube to limit oxygen diffusion during the microwave heating. The microwave reaction setup synthesis process is demonstrated in Fig. 1. A domestic microwave oven (1350 W Thomson, UK) was used to radiate the precursor for 30, 60, 80, and 100 s, then left to cool down to room temperature.

### Material characterization

The elemental composition of the produced NaFePO_4_/C is analyzed using an ICP-AES instrument (Leeman Labs Prodigy-USA), while carbon content is measured by an HCNS analyzer (ELEMENTAR-Micro VARIO Cube, Germany). Structural analysis is performed using Shimadzu-XRD-6000 Diffractometer, Japan, and Raman spectroscopy (SENTERRA, Bruker, Germany). The morphology is studied using scanning electron microscope (JEOL JSM- 6360 LA SEM, Japan), transmission electron microscope (JOEL, JEM 2100 plus TEM, Japan) attached with a Selected Area Electron Diffraction (SAED) unit, particle size analyzer (Beckman Coulter N5 submicron PSA, USA) and a surface area analyzer (BELSORP-miniX, Germany). For PSA analysis, the powder samples are prepared by dispersing 2 mg of powder in 5 ml of Di-methyl Sulfoxide (DMSO) and sonication in an ultrasonic path for 30 min.

### Electrochemical characterization

#### Electrode preparation

Electrodes are prepared by mixing active materials with conductive carbon (industrial quality graphite nanoplatelets, ACS material, USA) using ball milling at 400 rpm for 30 min. *N*-methyle pyrrolydone (NMP) (99.5%, Merck, Germany) containing 20 mg ml^−1^ PVDF (99%, Alfa Aesar, Germany) is added to the mixture. The final weight ratio of (active material:conductive carbon:PVDF) is (75:15:10). The slurry has been plated on Al foil for battery and on Cu foil for supercapacitor applications. The unloaded parts of the current collectors are covered with an isolating tape. The current collectors were weighed before and after material loading. The weight of conductive additive and binder are subtracted to determine the active mass loading in the electrodes.

#### Cell fabrication

Electrochemical cells for battery fabrication are composed of the working electrode as a cathode, sodium metal as both the counter and reference electrodes, fiberglass filter paper (GFD Wattman, UK) as a separator and 1 M NaClO_4_ (99% Across, Germany) in propylene carbonate (PC) (99%, Fluka, Germany) as the electrolyte. Figure 1 shows the synthesis, characterization and components of the used homemade Teflon coin cell. The cells are assembled inside a dry air-filled glove box instead of argon filled glove box, leading to further cost reduction. The symmetric electrochemical capacitors are assembled in air using a GFD separator soaked with saturated Na_2_SO_4_ (99% Fischer, Germany) sandwiched between the two similar electrodes.

#### Electrochemical measurements

Cyclic voltammetry, EIS, and charge–discharge tests were performed by two electrode cells using a workstation (Autolab. Aut87070, Germany). Charge–discharge and CV tests were conducted in the range between 1 and 4 V for batteries and between 0.0 and 1.1 V for supercapacitors. EIS measurements were conducted at the initial open circuit potential of the battery, with amplitude of 50 mV, and at a voltage of 50 mV for supercapacitors. The frequency range was from 1 MHz to 0.2 Hz.

## Results and discussion

### Materials characterization

#### Crystal structure and elemental analyses

Elemental analyses of all synthesized samples are shown in Table S1, giving an approximate atomic ratio of Na, Fe, and P to be nearly 3:2:3, indicating that most water removal and chemical reaction processes took place within the first 30 s of the microwave heating. This may be due to the high loss tangent value of SiC present in the rice straw ash^28,29^. The remaining carbon content present in samples is 5.4, 4.7, 4.1, and 3.8% for MW heated samples at 30, 60, 80,and 100 s respectively, indicating gradual loss of carbon content as the MW time increases from 30 to 100 s.

**Figure 2:**
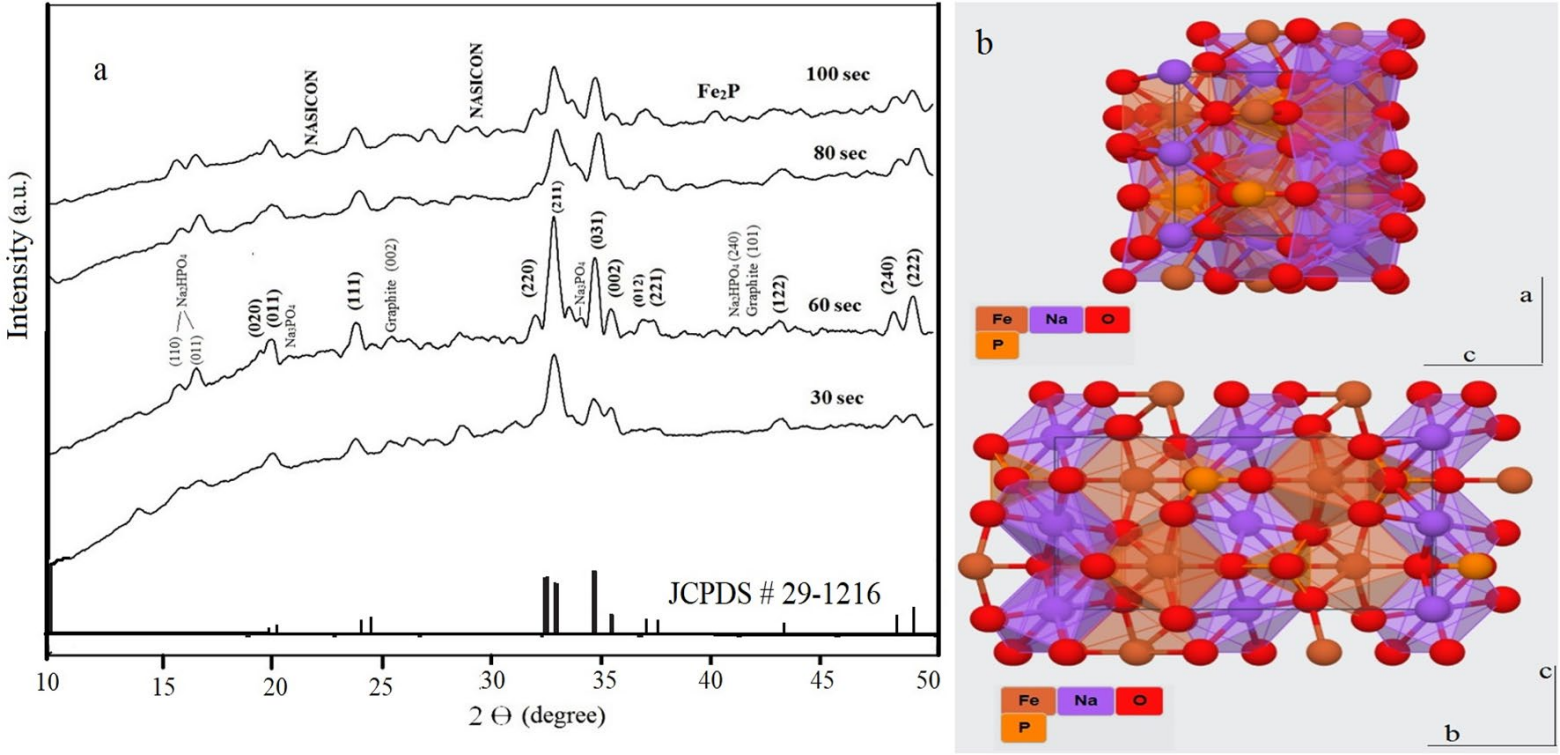
(**a**) XRD patterns of microwave solid state synthesized sodium iron phosphate samples, (**b**) structure of maricite NaFePO_4_.

The XRD pattern (Fig. 2) shows the formation of maricite phase NaFePO_4_ (JCPDS card #29-1216) after the first 30 s of the reaction. The maricite crystal structure is orthorhombic with the *pmnb* space group. The hump present in the graphs indicates a large amount of amorphous phase, which is calculated using Match 3 software according to Eq. (1)^30^:

1 $$\mathrm{\% Crystallinity }= (\mathrm{Area\,of\,crystalline}/\mathrm{Total\,area}) \times 100\mathrm{\%}$$

The degrees of crystallinities and crystallite sizes for all samples are summarized in Table1. They indicate the reduction of amorphous phase content with increasing MW time. The crystallite sizes of maricite phase for all samples are calculated from XRD data by applying Scherer’s equation on the (211) and (031) planes. The maximum maricite crystallite size (17.6 nm) is obtained the sample Fe(60). Originally, the reactants stoichiometry was designed for the preparation of NASICON sodium iron phosphate, but the maricite phase has been created beside unreacted species from precursor as Na_2_HPO_4_ (JCPDS # 35-0735) or by products as Na_3_PO_4_, (PDF#33-1272) according to Eq. (2):

2 $${\text{Fe}}_{{3}} \left( {{\text{PO}}_{{4}} } \right)_{{2}} \cdot{\text{8H}}_{{2}} {\text{O}} + {\text{NaH}}_{{2}} {\text{PO}}_{{4}} + {\text{Na}}_{{2}} {\text{HPO}}_{{4}} + {\text{glucose}} + {\text{ACB}} \to {\text{Na}}_{{2}} {\text{HPO}}_{{4}} \left( {{\text{unreacted}}} \right) + {\text{NaFePO}}_{{4}} - {\text{C}} + {\text{FePO}}_{{4}} - {\text{C}} + {\text{Na}}_{{3}} {\text{PO}}_{{4}} - {\text{C}} + {\text{H}}_{{2}} {\text{O}} \uparrow$$

**Table 1:** Crystallite sizes and DOC of NaFePO_4_-C composite samples.

Sample	FWHM (degree)		Crystallite size (Maricite) (Mean) nm	FWHM Graphite (002)	Crystallite size Graphite (nm)	Degree of crystallinity (%)
	(221) (Maricite)	(031) (Maricite)				
Fe(30)	0.71	0.74	12	0.78	10.92	20.48
Fe(60)	0.74	0.55	17.6	0.8	10.65	33.68
Fe(80)	0.55	0.45	12.3	1.25	6.8	41.09
Fe(100)	0.45	0.84	13	1.17	7.3	62.86

As the temperature increases with increasing MW time over 60 s, a part of the maricite structure reacts with the noninvolved phases (FePO_4_, Na_3_PO_4_) forming other crystalline phases as NASICON (PDF#045-0319) as shown in XRD patterns for Fe(80) and Fe(100) samples. The Fe_2_P phase usually appears with strong reductive conditions at higher MW durations. This phase has been reported to decrease the ionic conductivity by blocking alkali ion diffusion pathways in olivine LiFePO_4_^31^.

Graphitic structure starts to appear at 2ϴ around 26.35° and 43.07° corresponding to plans (002) and (101) respectively. With increasing MW duration to 60 s, the intensity of the (002) peak increased due to the graphitization of glucose with broadening and shifting to lower 2ϴ values. With more increase in temperature, graphite layers decrease due to consumption of the remaining diffused oxygen, which can be indicated by a decrease in calculated crystallite sizes of graphite for Fe(80) and Fe(100) samples as shown in Table 1.

Calculated unit cell parameters are listed in Table S2. The dimensions for Fe(60) sample are very close to the theoretical cell parameters of maricite phase. With increasing MW time, a reduction occurs along the *a* direction while a slight expansion in the *b* direction takes place. This may occur as a step for incomplete conversion to other phases present in the last MW time Fe(100) such as NASICON and Fe_2_P. Full conversion to pure NASICON phase during solid state preparation method has been reported at 750 °C for 12 h^32^.

#### Raman spectra

**Figure 3:**
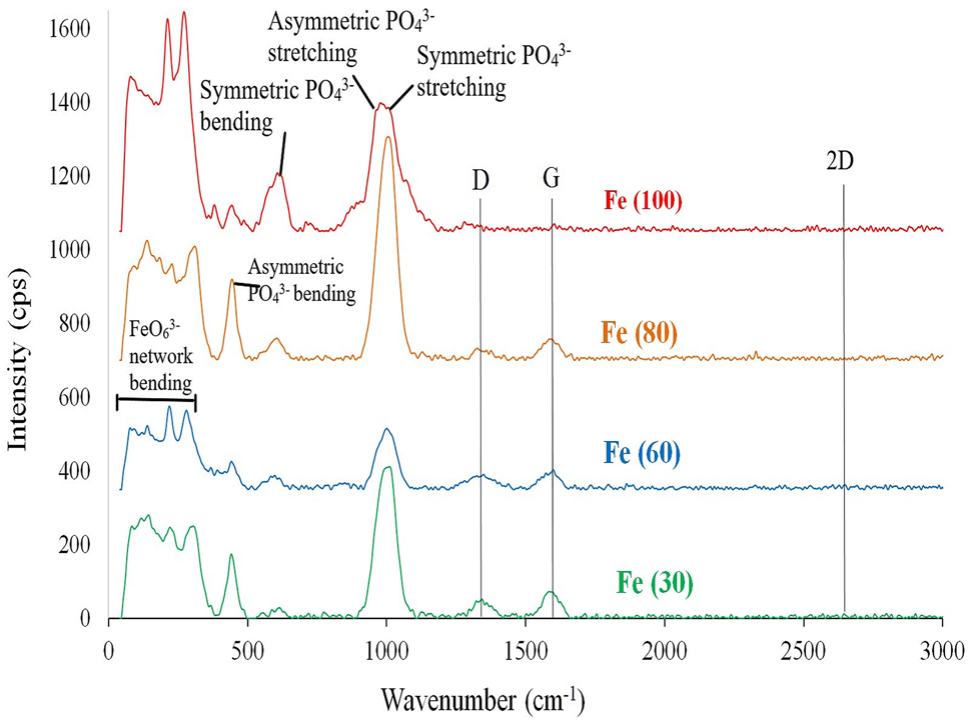
Raman spectra for microwave solid state synthesized sodium iron phosphate samples.

The Raman spectra of all the prepared samples are presented in Fig. 3.They show four peaks that are characteristic to the vibrational modes of phosphate group. The peaks at around 950 and 1000 cm^−1^are related to asymmetric and symmetric stretching respectively, while the peaks at around 450, and 600 cm^−1^ are related to asymmetric and symmetric bending respectively^33^. The appearances of fingerprint peaks in the region between 200 and 400 cm^−1^ are characteristic to maricite FeO_6_^3−^ network bending^33^. Also, the peaks at around 1350, 1600, and 2650 cm^−1^ are related to D, G, and 2D bands characteristic to carbon coating^34^. Their numerical intensities decrease as the microwave time increases, which may be due to the consumption of carbon content upon heating. A partial graphitization might take place, forming graphene clusters inside amorphous carbon coating parts. This is indicated by the decrease in the I_D_/I_G_ ratio to its minimum value of 0.56 at a MW time of 80 s as shown in Table 2. Also, the number of defects may be assumed from the calculation of the ratio of I_2D_/I_G_. It gives its minimum value of 0.1 for sample Fe(80) with a maximum crystallite size of 30 nm and the lowest defect content. The crystallite size (L_a_) of graphene clusters in nm is calculated by Eq. (3)^35^:

3 $${L}_{a }\left(nm\right)=\left(2.4 \times {10}^{-10 }\right){\lambda }^{4 }{(\frac{{I}_{D }}{{I}_{G}})}^{-1}$$

where λ is the laser wavelength excitation length of 514.5 nm.

**Table 2:** Values of D, G and 2D bands and their related intensities with a comparison of I_D_/I_G_ and I_2D_/I_G_ for MW heated samples.

Samples	D (cm^−1^)	ID (cps)	G (cm^−1^)	IG (cps)	I_D_/I_G_	2D (cm^−1^)	I_2D_ (cps)	I_2D_/I_G_	L_a_ (nm)
Fe(30)	1343	52.2	1584	73.7	0.72	2645	12.75	0.17	23.3
Fe(60)	1348.5	41	1599	53.71	0.76	2647.5	13.03	0.24	22.1
Fe(80)	1320	32.3	1587	97.97	0.56	2647.5	5.71	0.1	30
Fe(100)	1326	16.4	1602	19.97	0.82	2652	4.66	0.23	20.5

#### Morphology analysis

**Figure 4:**
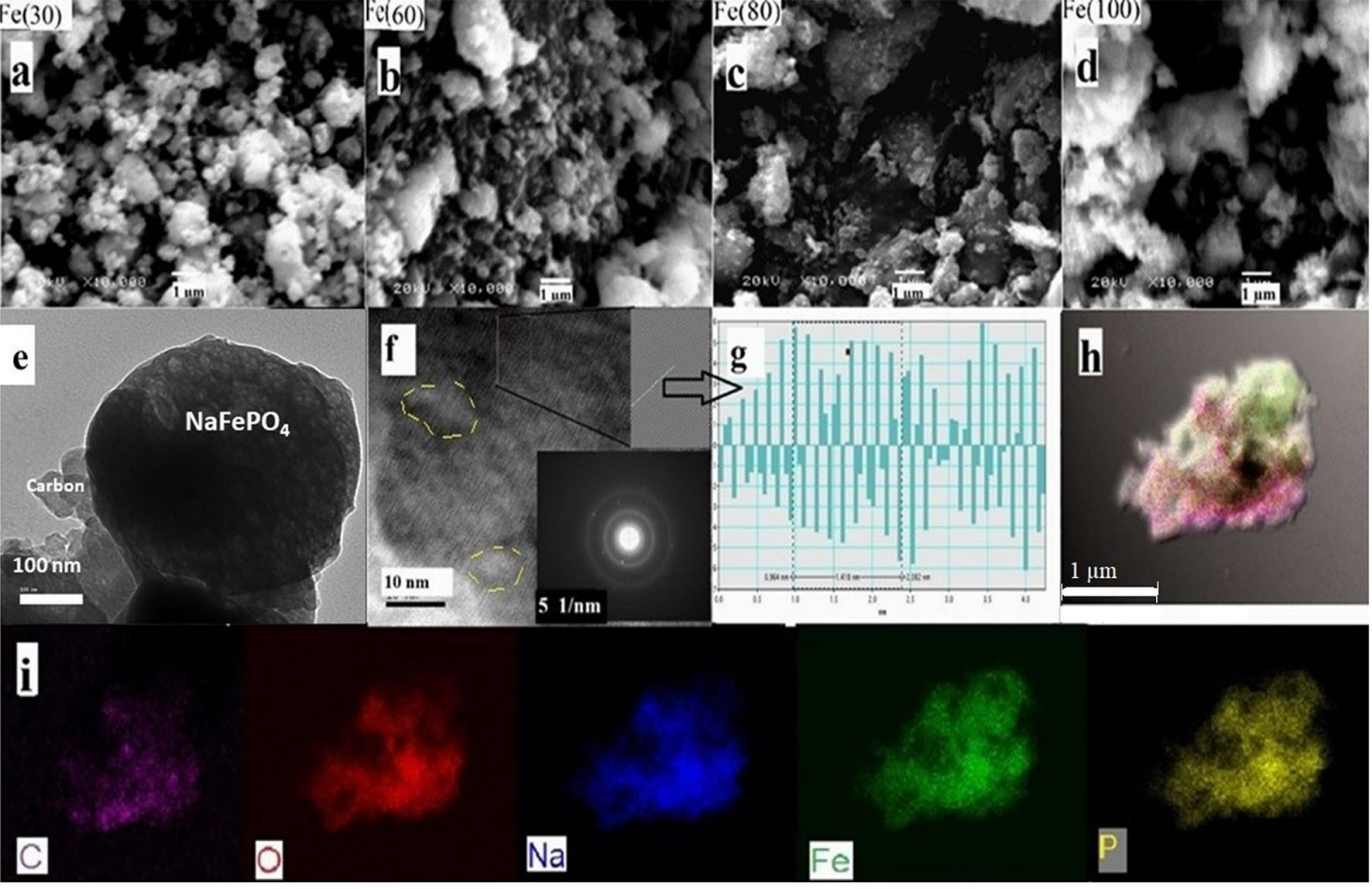
(**a**–**d**) SEM images at ×10,000 for Fe(30), Fe(60), Fe(80) and Fe(100) respectively. (**e**) Low magnification TEM image showing carbon compositing with NaFePO_4_ particles, (**f**) High magnification TEM image and SAED image for NaFePO_4_ spot, (**g**) FFT images and IFFT profile of Fe(60) for selected area along (240) plane and (**h**,**i**) Elemental mapping showing C, O, Na, Fe, and P distribution on the selected particle.

Figure 4 represents a general illustration for SEM, TEM and elemental mapping for samples. Figure 4a–d shows SEM images for all the prepared samples at different MW durations, which possess randomly shaped and sized particles. This could be considered as a common feature for solid stat methods^12^. Particle size analysis (PSA) shows a wide particle size distribution curve. The mean particle sizes calculated from SEM and PSA are presented in Table 3. Sample Fe(60) has the lowest average size (around 200 nm for PSA and SEM). Surface area of particles measured by BET shows a gradual decrease with increasing MW time due to the graphitization and combination of high surface area ACB particles with sodium iron phosphate particles (Vulcan XC 72 R has a typical surface area of 235 m^2^ g^−1^^36^). The measured surface areas and pore diameters are listed in Table 3. The BET and BJH plots are provided with supporting information Figures S2 and S3. The surface area for Fe(60) is 10.1 m^2^ g^−1^.The previously reported surface areas for NaFePO_4_ are range from 3.5 for micro particles to 120 m^2^ g^−1^ for 15 nm hollow nanoparticles^16,37–39^. TEM images for the Fe(60) sample show that the graphitized carbon is well distributed on the particle’s surface, but with unequal thickness. The large particles observed in TEM images are composed of small irregular spherical nano-crystallites of about 10–15 nm with an intergranular amorphous matrix as shown in Fig. 4e,f. Selected area electron diffraction (SAED) is shown in the inset of Fig. 4f confirms the presence of a large amount of amorphous phases indicated by continuous circles and a low amount of crystalline phase indicated by plane spots^39^. Figure 4f,g represent the fast furrier transform (FFT) image and Inverse FFT (IFFT) profile for the selected area, giving d spacing of 1.77 Å for Fe(60) sample along (240) plan. They also indicate the presence of many lattice defects and different growth directions due to the fast microwave heating. Figure 4h,i represent the elemental mapping for the Fe(60) sample, showing the uniform distribution of Na, Fe, P, and O in the selected particles. Carbon coating was also successful and nearly homogenous along the particle’s surface. This homogeneity may be due to the very low size of activated carbon used and the presence of glucose as an additional carbon source.

**Table 3:** Mean particle sizes of microwave synthesized samples measured by PSA and manually from SEM.

Sample	SEM mean (nm)	PSA mean (nm)	Surface area (m^2^g^−1^)	Pore diameter (nm)
Fe(30)	273	266.2	11.96	73.55
Fe(60)	218.3	193.8	10.1	30.1
Fe(80)	331	347.3	8.568	34.3
Fe(100)	420	546	6.2465	33.3

### Electrochemical characterization

#### Sodium ion battery cell

**Figure 5:**
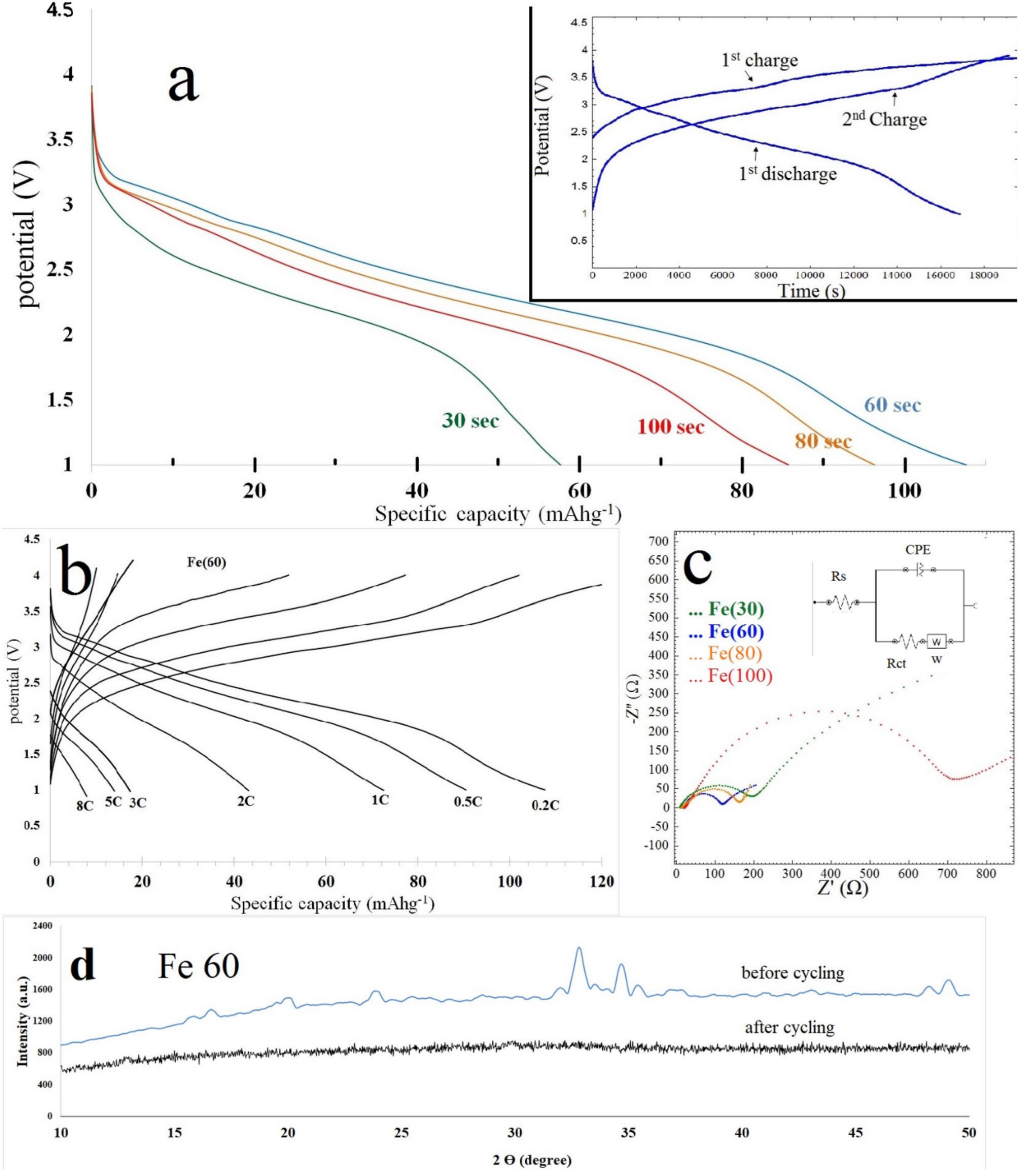
(**a**) First discharge curves showing discharge capacities (at 0.2 C rate) for samples prepared by microwave heating at different times, side graph is the first charge–discharge and 2nd charge cycles at 0.2 C rate for samples Fe(60), showing the first high energy electrochemical sodium extraction process, (**b**) charge–discharge curves for Fe(60) sample at different C-rates, (**c**) Nyquist plots of samples prepared at different MW times, side graph shows the equivalent circuit, (**d**) the XRD patterns of Fe(60) before and after cycling.

Figure 5a shows the first discharge profiles of samples at 0.2 C rate. The discharge capacities at different C-rates are listed in Table 4. The best discharge capacity obtained is 108.4 mA h g^−1^ for Fe(60) sample Vs Na/Na^+^, which is characterized by the presence of the highest amount of crystalline maricite phase (according to XRD patterns) and the smallest particle size (according to PSA). The order of capacity was Fe(60) > Fe(80) > Fe(100) > Fe(30). For the sample Fe(30), the low capacity may refers to the presence of a large amount of unreacted inactive raw materials. The decrease in capacity with increasing microwave time above 60 s may refer to the decrease in particle size and to the increase in surface area (Table 3). The first charge cycle for all samples exhibits a relatively elevated voltages due to energy consumed during extraction of Na that occupies the corners of the orthorhombic bipyramid in the maricite structure^40^. As shown in the inset of Fig. 5a, the second charge processes have lower voltages compared to the first. This is because the intercalated Na ions occupy the framework channels in maricite NaFePO_4_. After the first charge–discharge cycle, maricite NaFePO_4_ is converted to amorphous phase, so that Na^+^ ions become easier to extract^12,13^. Figure 5d shows the difference in XRD pattern before and after cycling, indicating the conversion of the maricite into an amorphous phase. Nyquist plots of samples are presented in Fig. 5c. The R_CT_ values indicate that the sodium ion diffusivity inside the cathode matrix was in the order of Fe(60) > Fe(80) > Fe(100) > Fe(30). Sodium ion diffusion coefficients (D_Na_) are calculated from the low frequency region according to equation S2 (Supplementary). The calculated values are listed in Table 4. The rate capabilities of samples followed their R_CT_ order, indicating that the enhancement in electronic conductivity of samples is the key factor for improving rate capability. Figure 5,b shows the charge–discharge curves at a different rates for sample Fe(60), the corresponding capacities for other samples are listed in Table 4. Generally, iron based sodium ion batteries suffer from capacity fading with increasing discharge rate^41^.

**Table 4:** Specific capacities of microwave prepared samples at different current rates.

Sample	Specific capacity mA h g^−1^ at					Capacity retention at 2 C (%)	Warburg factor (K)	Diffusion coefficient of Na^+^ (D_Na_) cm^2^ s^−1^	R_CT_
	0.2 C	0.5 C	1C	2C	3C +				
Fe(30)	57.7	31.13	24.53	16.93	–	29.35	177.75	3.9 × 10^–15^	184.6
Fe(60)	108.44	90.47	72.5	43.18	17.35 13.9 (5C) 7.9 (8C)	40.13	28.464	1.5 × 10^–13^	105
Fe(80)	96.33	80.69	65.93	35.2	23	36.54	25.062	1.9 × 10^–13^	149.7
Fe(100)	82.97	67.5	49.86	27.79	18.88	33.5	75	2.18 × 10^–14^	702.3

#### Supercapacitors cell

**Figure 6:**
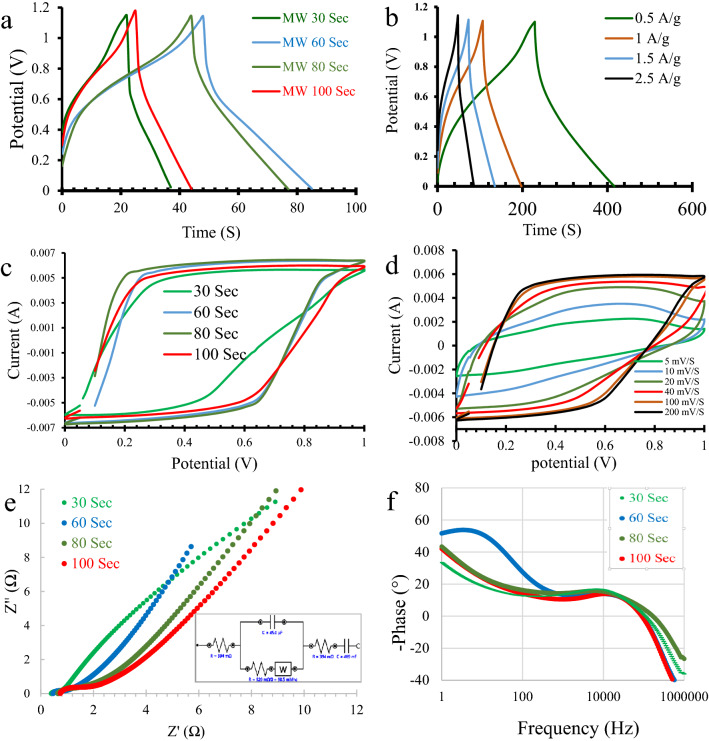
(**a**) charge discharge curves for samples prepared at different MW durations at 2.5 A g^−1^, (**b**) charge discharge curves for Fe(60) sample at different currents, (**c**) CV curves for samples prepared at different MW times at 200 mV S^−1^, (**d**) CV curves for Fe(60) sample at different scan rates, (**e**) Nyquist plot for samples prepared at different MW durations in an electrochemical capacitor cell. The inset represents the supercapacitor’s equivalent circuit (**f**) bode phase plot for samples prepared at different MW durations in an electrochemical capacitor cell.

Figure 6a shows the galvanic charge discharge curves for prepared materials as symmetric supercapacitor electrode. The value of calculated capacities at discharge currents of 0.5, 1, 1.5, and 2.5 A g^−1^ are listed in Table 5. The calculations of specific capacitance (C), energy density (E), and power density (P) are based on Eqs. (4), (5), and (6), respectively^42,43^:

4 $$\mathrm{C}= \frac{I \Delta t}{m \Delta V}$$

5 $$\mathrm{E}=\frac{\mathrm{C }\times\Delta {\mathrm{V}}^{2}}{2 \times 3.6}$$

6 $$\mathrm{P}=\frac{\mathrm{I}_{\text{max}}\times \mathrm{ \Delta V}}{2 \mathrm{m}}$$

where I is the constant discharge current (A), Δt is the discharge time (sec.), ΔV is the operating potential window (V), m is the mass of the loaded active material on one electrode (g) and I_max_ is the maximum current used for discharge. The optimized microwave time for this procedure is 60 s. Theoretically, its preferable particle size and surface area are the main factors leading to its high capacity as a supercapacitor. Figure 6b shows the charge–discharge curves of Fe(60) sample at different currents. The values of capacitance show no or little decrease with increasing working current up to 2.5 A g^−1^ in all samples. This behavior was unexpected for a sodium intercalation material because both mechanisms of insertion-extraction and electrical double layer (EDL) formation should be responsible for charge storage. Here, it is suggested that only the EDL mechanism was included, while the intercalation does not take place because the current densities applied to supercapacitor cells are very high compared to those applied to battery cells (0.2 C-rate is equal to 0.023 A g^−1^). Figure 6c,d show the capacitive behavior for samples where the area inside the CV curve is indicative of capacitance, (C = Area under the CV curve/2(scan rate × ΔV × m))^44^. However, the calculations using charge discharge curves are easier and more accurate.

**Table 5:** Specific capacitances of microwave prepared samples at different currents.

Sample	Specific capacitance (F g^−1^) at				Power density (W kg^−1^) at 2.5 A g^−1^	Energy density (Wh kg^−1^) at 0.5 A g^−1^
	0.5 A g^−1^	1 A g^−1^	1.5 A g^−1^	2.5 A g^−1^		
Fe(30)	55.5	48.2	40.9	40	678.5	9.33
Fe(60)	86.3	85	85	82.7		14.5
Fe(80)	77.2	73.6	73	70.45		13
Fe(100)	43.6	42.3	41.5	40.9		7.33

EIS analysis for samples as symmetric supercapacitors shows a small semicircle at the high frequency region (Fig. 6e). The plots confirm the preferable kinetics for Fe(60) sample, since it has the lowest R_CT_ at the high frequency region, the highest slope of straight line at the low frequency region of the Nyquist plot, and the highest phase angle (Fig. 6f), (the ideal supercapacitor theoretically shows a perpendicular line on the Nyquist plot and a phase angle of 90°)^42^.

**Figure 7:**
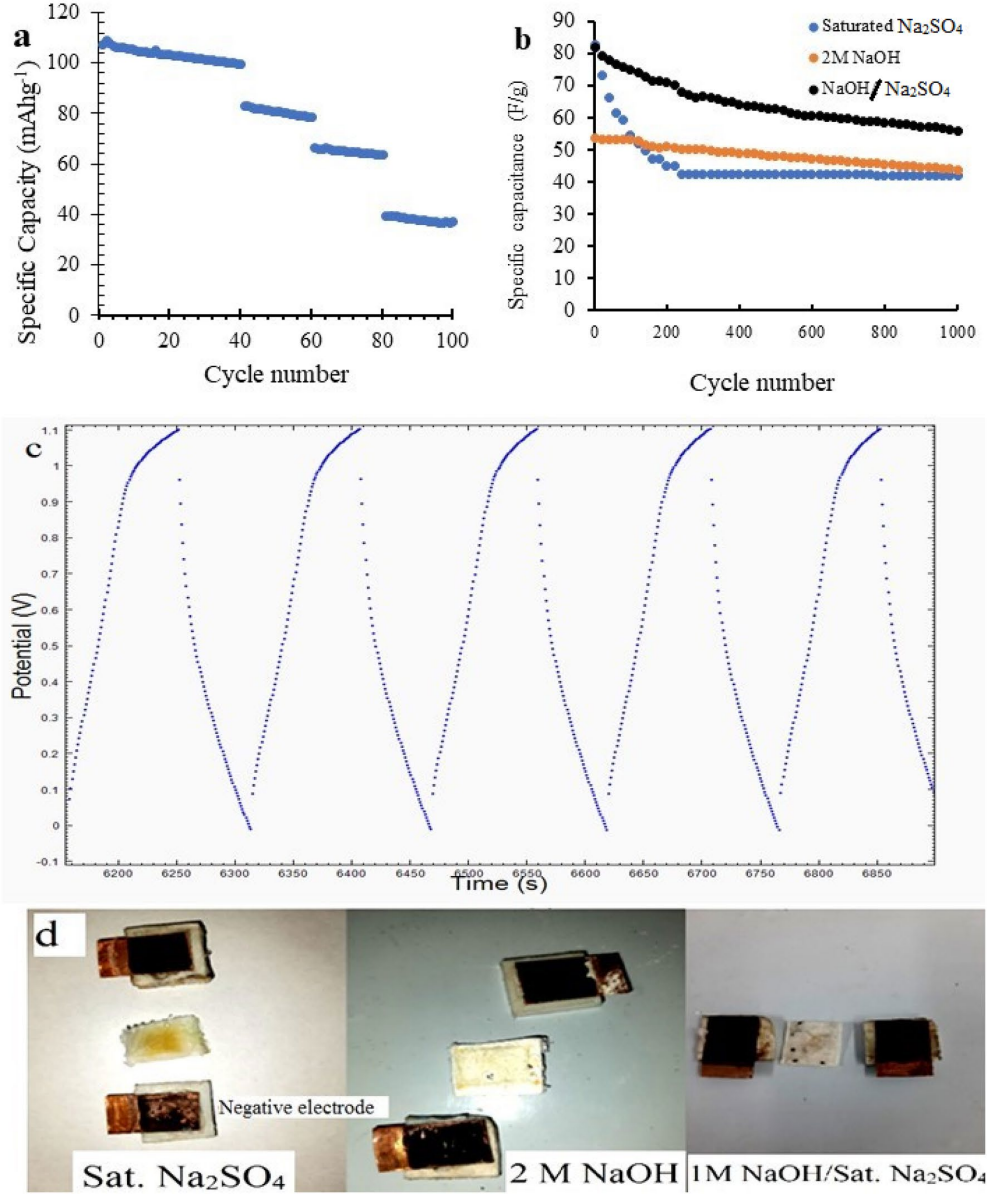
(**a**) Cyclic performance of Fe(60) sample in Na-ion cell at different C rates. (**b**) Cyclic performance of Fe(60) sample as symmetric electrochemical capacitor in different electrolytes, (**c**) cycles 50–55 of Fe(60) sample cycling test with saturated Na_2_SO_4_ and (**d**) electrodes after 1000 cycles using different electrolytes.

The cycling performance of Fe(60) sample was measured as a battery for 100 cycles (Fig. 7a) and as an electrochemical capacitor for 1000 cycles at 2.5 A g^−1^ (Fig. 7b). As a sodium ion battery, the specific capacity was slightly increased in the 2nd and 3rd cycles. This increase may be due to conversion to an amorphous phase^13^. The capacity retention was 92.85% after 40 cycles at 0.2 C, 94.7% after the next 20 cycles at 0.5 C, 95.6% after the next 20 cycles at 1 C and 93.5% after the last 20 cycles at 2 C (relative to the capacity of the first cycle at the c-rate start). As an electrochemical capacitor, the capacity retention after 1000 cycles was only 41.8%, and most of the capacitance loss occurs in the first 200 cycles.

It was noticed that the active material is eroding in the neutral electrolyte that becomes slightly acidic at the negative electrode^45^. The acidic media increase the solubility of iron phosphates. Formation of iron III sulfate and sodium phosphate in electrochemical reaction is another possible reason for dissolution as an explanation for cathode material erosion. Figure 7d shows the difference between positive and negative similar electrodes after 1000 cycles using saturated Na_2_SO_4_, indicating gradual dissolution of active material at the negative electrode in the electrolyte. The cycling test was conducted using 2 M NaOH and a mixture of 1 M NaOH with saturated Na_2_SO_4_ as electrolyte. The active material shows more stability in the two basic electrolytes as shown in Fig. 7c and enhanced cycle retention, indicating that the acidic dissolution mechanism is more likely responsible for active material corrosion. For 2 M NaOH cells, the recorded initial capacitance was only 53.6 F g^−1^ at current 2.5 A g^−1^ and about 81.7% of capacity was retained after 1000 cycles. For NaOH/Na_2_SO_4_ cells, an initial capacitance of 81.8 F g^−1^ is obtained with capacity retention of 68.3% after 1000 cycles at 2.5 A g^−1^.

When comparing battery performance to other works, this work is in the middle region of the recorded capacities and rate capabilities. On the other hand, the time and cost of energy consumed for synthesis are incomparable. Table S3 summarizes a comparison between our study and other prepared cathode materials. The comparison includes the difference in preparation methods and time consumed for each preparation step.

When comparing electrochemical capacitor performance, we concluded that the capacitance does not increase due to the intercalation ability of maricite/amorphous sodium iron phosphate, resulting in uncompetitive capacity. In addition, this work requires further enhancement in cyclic performance and durability of active materials. However, the investigation of supercapacitive ability was limited to Ni and Co phosphates^18^. To the best of our knowledge, this is the first report that uses this method for synthesis of maricite/amorphous NaFePO_4_ and the first report that investigates NaFePO_4_ as a symmetric electrochemical capacitor.

## Conclusions

Sodium iron phosphate–carbon nanocomposites were prepared by an ultrafast microwave technique. Egyptian rice straw ash as MW absorber enabled the synthesis in a very short time and with low cost process. This fast MW heating with the presence of slightly reductive conditions replaced the need for argon gas in the synthesis, crystallization, and carbon coating processes, which are done in a one step. In addition, this method has a high degree of flexibility to use many sodium, transition metal, phosphate, and carbon sources, which enables many available modifications and optimization factors to enhance capacity, rate capability, and cyclic performance. The downside of this method is the irregular and non-uniform shape and size of the produced particles. The materials produced by the microwave are a polymorphic NaFePO_4_ with a small crystallite size. The fast heating does not allow the crystal growth to be completed and the 10–15 nm particle size is obtained, as evidenced by TEM. However, the particles tend to agglomerate, forming larger grains with an average size of about 300 nm, which limits the sodium ion diffusivity. So, the produced active materials showed good electrochemical activity as a battery with a capacity of 108.4 mA h g^−1^, with a moderate rate capability, and good cyclic stability. As a supercapacitor, the capacitance was 86.3 F g^−1^ and showed cyclic stability in alkaline electrolytes with capacity retention of 81.7% of capacity after 1000 cycles at 2.5 A g^−1^, which could be useful in short-term general applications.

## Supplementary Information

Supplementary Information.

## Data Availability

All data generated or analysed during this study are included in this published article [and its supplementary information files].
