# Supplementary material for: Ultra-fast green microwave assisted synthesis of NaFePO4-C nanocomposites for sodium ion batteries and supercapacitors
Source: Sci Rep. 2022 Sep 29;12:16307. doi: 10.1038/s41598-022-20329-x (PMC9522881; doi:10.1038/s41598-022-20329-x)
Supplement: Supplementary file 1 — Supplementary Information. [file 41598_2022_20329_MOESM1_ESM.docx]

**Ultra-fast Green Microwave Assisted Synthesis of NaFePO­_4_-C Nanocomposites for Sodium Ion Batteries and Supercapacitors**

**Wael Wazeer ^1*^, MarwaM. Nabil^1^, Mohamed Feteha^2^,Moataz B. Soliman^2^, Abd El-Hady B. Kashyout^1*^**

**Supporting information**

- **Preparation of Fe_3_(PO4)_2_.8H_2_O**

The precursor of Fe_3_(PO4)_2_.8H_2_O was prepared by dissolving 1.2 M of FeSO_4_.7H_2_O (Riedel-de Haen Germany) with 3g of glucose in 800 ml of de-ionized water. 0.8 moles (54.8 ml) of phosphoric acid 85% (Riedel-de Haen, Germany) was added dropwise to the solution and stirred for 10 min. The pH of the resulted solution raised to> 9 with the addition of 5 M NaOH solution (99%, Carl Roth GmbH, Germany).The solutions are mixed under continuous stirring until the grey color of Fe_3_(PO_4_)_2_.8H_2_O appears. Finally, the powder was filtered and washed twice using DI (deionized water) to remove soluble Na_2_SO_4_ and then dried overnight at 50 ^o^C or lower temperature to prevent oxidation of Fe^+2^ to Fe^+3^. The ICP analysis is performed to check the chemical structure of Fe_3_(PO_4_)_2_.8H_2_O by dissolving 10 mg of powder in 50 ml 0.1 molar HCl. The concentration of iron is 66.3 ppm while phosphorus is 24.5 ppm, the atomic ratio of Fe:P is 3:2. The concentration of Na is lower than the detection limit of 0.2 ppm, which means high purity of the precusor. Figure S1 represents the XRD pattern for produced Fe_3_(PO4)_2_.8H_2_O precursor material. The pattern shows the main peak at (2θ = 13.6^o^) and other related peaks which is related to the peaks present in JCPDS No. 01-079-1928 for the vivianite Fe_3_ (PO4)_2_.8H_2_O.

Figure S1: XRD pattern of prepared Fe_3_(PO4)_2_.8H_2_O.

**Table S1: Elemental analyses of MW synthesized NaFePO_4_-C nanocomposites and their precursor mixtures**

| **Microwave**  **Time**  (Second) | **Na** | | **Fe** | | **P** | | **O^*^** | | **C - content Wt %** |
| --- | --- | --- | --- | --- | --- | --- | --- | --- | --- |
|  | **Wt. %** | **At. %^**^** | **Wt.%** | **At. %^**^** | **Wt. %** | **At. %^**^** | **Wt. %** | **At. %^**^** |  |
| **0**  **(Precursor)** | 10.6 | - | 17.7 | - | 15.2 | - | 48.5 | - | 8 |
| **30** | 14.08 | 14.81 | 22.71 | 10.14 | 18.81 | 14.9 | 38.87 | 60.15 | 5.41 |
| **60** | 14.1 | 14.83 | 22.74 | 10.19 | 18.96 | 14.84 | 39.18 | 60.14 | 4.72 |
| **80** | 14.19 | 14.76 | 22.91 | 10.27 | 19.1 | 14.81 | 39.47 | 60.16 | 4.06 |
| **100** | 14.23 | 14.9 | 22.98 | 10.59 | 19.14 | 14.79 | 40.55 | 59.72 | 3.83 |

**Table S2: Lattice constants of the prepared NaFePO_4_-C samples.**

| Sample | 2ϴ (degree) | d (A) | a | b | c | *hkl* |
| --- | --- | --- | --- | --- | --- | --- |
| Fe (30) | 19.96 | 4.44 | 7.03 | 9.24 | 5.07 | (011) |
|  | 31.96 | 2.8 |  |  |  | (220) |
|  | 35.38 | 2.53 |  |  |  | (002) |
| Fe (60) | 19.92 | 4.45 | 6.99 | 9.33 | 5.07 | (011) |
|  | 31.96 | 2.81 |  |  |  | (220) |
|  | 35.4 | 2.53 |  |  |  | (002) |
| Fe (80) | 19.90 | 4.46 | 6.07 | 9.56 | 5.04 | (011) |
|  | 48.46 | 1.88 |  |  |  | (240) |
|  | 35.6 | 2.52 |  |  |  | (002) |
| Fe (100) | 19.94 | 4.45 | 6.38 | 9.36 | 5.06 | (011) |
|  | 48.18 | 1.89 |  |  |  | (240) |
|  | 35.47 | 2.53 |  |  |  | (002) |
| Theoretical Maricite value |  | | 6.86 | 8.99 | 5.05 |  |

- Surface area and pore size plots


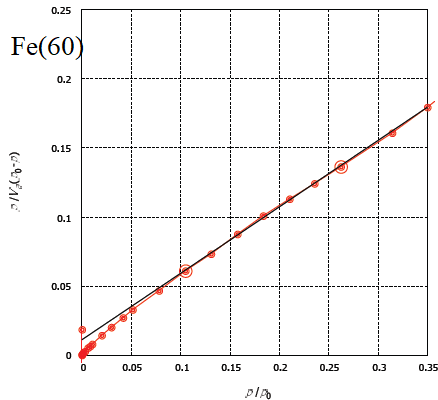

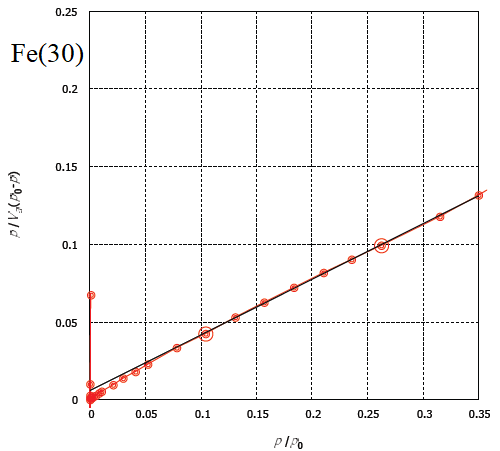

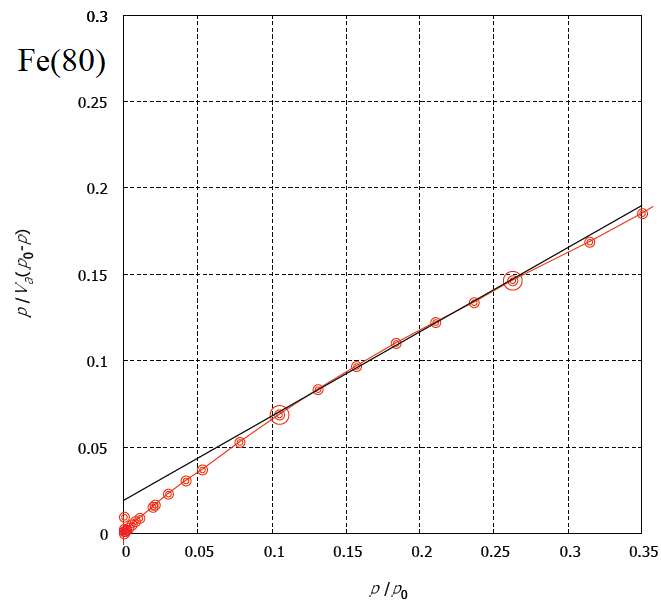

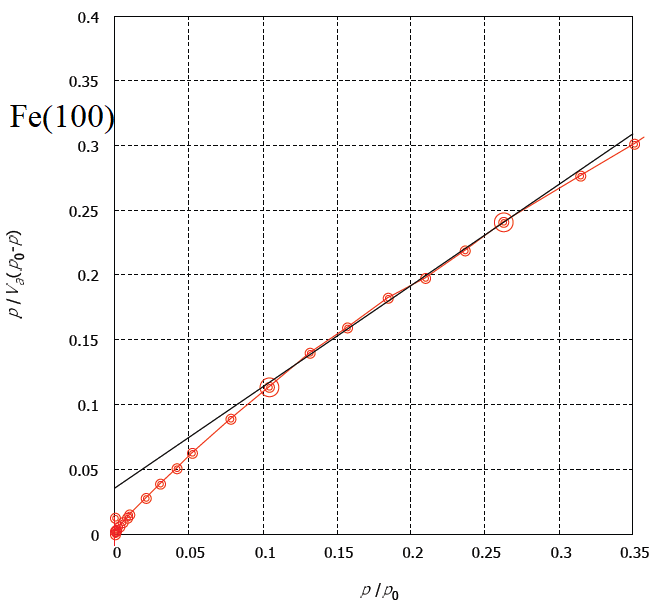


**Figure S2: BET plots for prepared samples at different MW durations.**


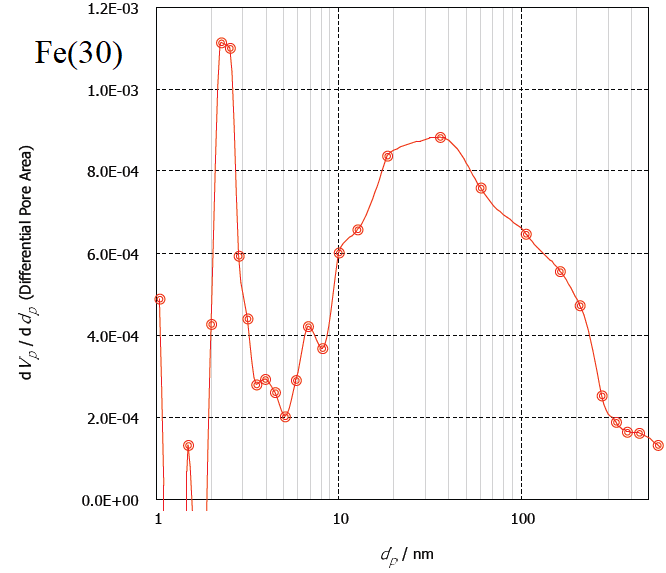

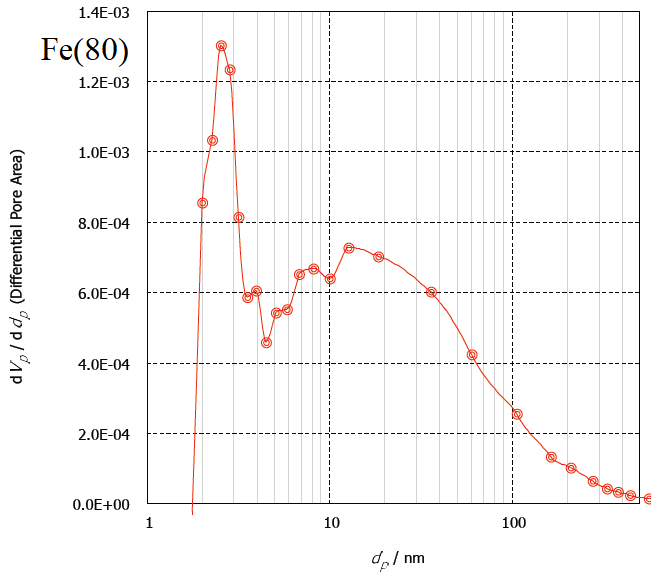

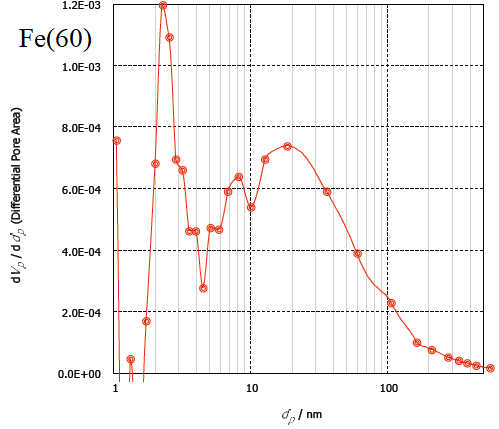

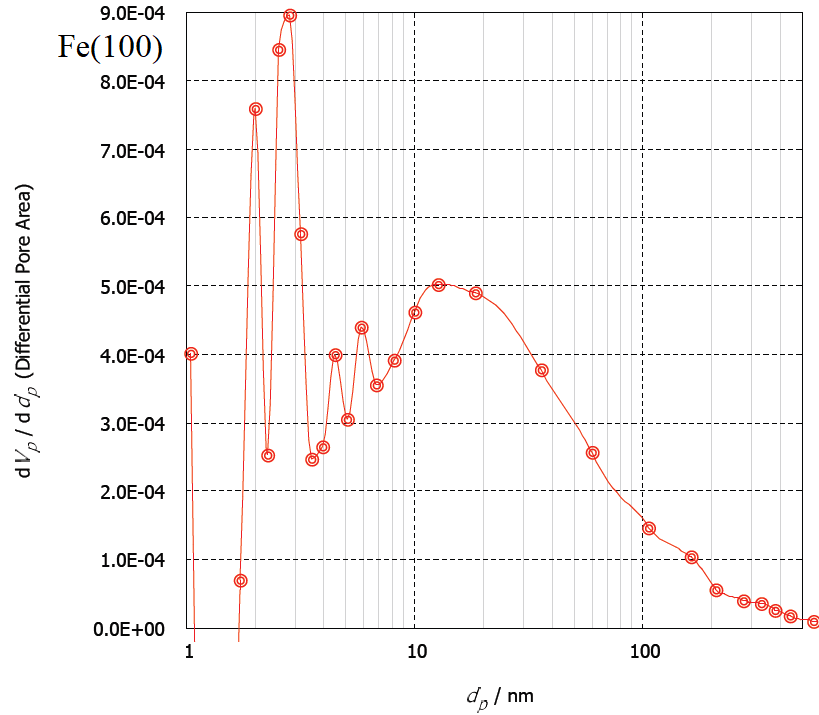


**Figure S3: BJH plots for prepared samples at different MW durations.**

- **Calculation of sodium ion diffusivity**

Z_W_ is the Warburg impedance,which is related to the Warburg factor by the equation:

Z_w_ = R_S_ + R_CT_ + *k* ω^-1/2^ (1)

Where ω is the radiant frequency and *k* is the Warburg factor (the slope of the straight line resulting from plotting ω^-1/2^ versus Z' values at the low frequency part of the impedance).

The diffusion coefficient of Na^+^ ion (D_Na+_) cm^2^/s is dependent on the overall ionic conductivity of active particles, conductive carbon additive, binder, and soaked electrolyte. It can be calculated from the equation:[S1]

D_Na+_ = R^2^T^2^ / 2 n^4^*F*^4^A^2^C^2^ *k*^2^ (2)

Where R is the gas constant (8.3145 *J*.mol^-1^.K^-1^), T is the absolute temperature, A is the surface area of the electrode, n is the number of electrons per molecule, F is the Faraday constant (96485.3329 A.s.mol^-1^), and C is the concentration of Na ions in the active material (1.925 x 10^-2^mol/cm^3^) dependent on elemental analysis and the density of sodium iron phosphate.

**Table S3: Comparison for obtained capacities and preparation methods for different cathode materials.**

| **Reference** | **Specific capacity (mA h g^-1^)**  **at 0.2 C**  **/ theoretical capacity** | **Synthesis method** | **Active material , Phase and (morphology)** | **No** |
| --- | --- | --- | --- | --- |
| This work | 107.6/154.2 | Ball mill at 500 rpm for 30 min and microwave heating for 60 Sec with rice straw ash | Amorphous /Maricite NaFePO_4_-C  (Nano/micro particles) | 1 |
| [S2] | 145/154.2 | Electrospinning of precursor with PVP for 48 h followed by heating for 2h at 300 ^O^C then for 2h at 600 ^O^C under high purity nitrogen | Maricite NaFePO_4_-C  (Nanowires) | 2 |
| [S3] | 115 (at 1C)/154.2 | Dissolution of soluble precursor and drying for 12 h at 160 ^O^C then heat treated in air at 300 ^O^C for 4h then under argon at 600 ^O^C for 10 h, and final ball milling at 800 rpm for 15 h | Amorphous NaFePO_4_-C  (Nanoparticles) | 3 |
| [S4] | 132/154.2 | Ball milling precursor at 500 rpm for 24 h, then heated at 350 ^O^C under Ar for 5 h, the powder pressed to pellet and reheated at 600 ^O^C under Ar | Amorphous /Maricite NaFePO_4_-C  (Nanoparticles) | 4 |
| [S5] | 147.8/155.2 | Heating organic precursor (Fe II stearate and Na Oleate) with NaH_2_PO_4_ at 380 ^O^C for 30 min under Ar/H_2_ gas, washing with water/ethanol and drying at 100 ^O^C for 12 h | Amorphous NaFePO_4_-C  (Hollow particles) | 5 |
| [S6] | 40/155.2 | Hydrothermal heating of dissolved precursors at 150 ^O^C for 6 h | Pure Maricite  NaFePO_4_  (Nanorods) | 6 |
| [S7] | 106/120 | Sol gel precipitation of Na and Fe acetates with (NH_4_)_3_PO_4_ followed by annealing at 500 ^O^C for 24 h under high purity argon | Na_4_Fe_3_(PO_4_)_2_(P_2_O_7_)/C  NASICON  (Nanoparticles) | 7 |
| [S8] | 106 (at 0.23 C)/131.7 | Solvothermal heating of dissolved precursors in ethylene glycol at 180 ^O^C for 10 h. Carbon coating made by ball milling annealing at 450 ^O^C for 2 h under argon | Na_0.71_Fe_1.07_PO_4_/C  (New crystal structure) (Nanorods) | 8 |
| [S9] | 60/99 | Dissolving precursors with citric acid (carbon source), then drying at 80 ^O^C, heating in an inert gas at 200 ^O^C for 12 h and at 350 ^O^C for 24 h | NaFe(SO_4_)_2_  Eldfellite  (Micro flakes) | 9 |
| [S10] | 98/107  (high operating voltage 4.2 V) | Dissolving organic precursor with citric acid (carbon source) then drying at 100 ^O^C, ball milling followed by heating at 750 ^O^C for 12 h under argon | Na_3_MnZr(PO_4_)_3_/C  NASICON  (Nanoparticles) | 10 |
| [S11] | 150/175 (based on 3 Na^+^ insertion /extraction) | Dissolving precursor and stirring at 80 ^O^C for 12 h, heating in air at 350 ^O^C for 4 h, and sintering at 800 ^O^C for 10 h under Ar. | Na_3_V_1.5_Cr_0.5_(PO_4_)_3_  NASICON  (Micro particles) | 11 |
| [S12] | 129 (at 0.1 C) /276  210 mAhg^-1^  at 0.1 C in presence of vinylene carbonate (VC) co electrolyte | Dissolving Tetraethyl ortho silicate, Mn acetate and citric acid in ethanol/water, stirring for 24 h, evaporation of ethanol and sintering at 750 ^O^C for 6 h under argon, ball milling with sodium carbonate at 500 rpm for 30 min, and 2^nd^ heating at 750 ^O^C for 8 h under Ar. Followed by 2^nd^ ball milling with conductive carbon at 500 rpm for 4 min. 3^rd^ annealing at 750 ^O^C for 8 h under Ar. | Na_2_MnSiO_4_/C Monoclinic  (Nanoparticles) | 12 |
| [S13] | 161/177 | Mn citrate and dihydroxybis(ammonium lactato)titanium was dissolved with NaH_2_PO_4_ and spray dried at 220 ^O^C followed by annealing at 650 ^O^C for 4 h under Ar. | Na_3_MnTi(PO_4_)_3_/C  NASICON  (Micro particles) | 13 |

**References**

[1] H. Li, C. Wu, Y. Bai, F. Wu, and M. Wang, “Controllable synthesis of high-rate and long cycle-life Na3V2(PO4)3 for sodium-ion batteries,” *J. Power Sources*, vol. 326, pp. 14–22, 2016, doi: 10.1016/j.jpowsour.2016.06.096.

[2] Y. Liu, N. Zhang, F. Wang, X. Liu, L. Jiao, and L. Z. Fan, “Approaching the Downsizing Limit of Maricite NaFePO4 toward High-Performance Cathode for Sodium-Ion Batteries,” *Adv. Funct. Mater.*, vol. 28, no. 30, 2018, doi: 10.1002/adfm.201801917.

[3] F. Xiong *et al.*, “Revealing the atomistic origin of the disorder-enhanced Na-storage performance in NaFePO 4 battery cathode,” *Nano Energy*, vol. 57, pp. 608–615, 2019, doi: 10.1016/j.nanoen.2018.12.087.

[4] J. Kim *et al.*, “Unexpected discovery of low-cost maricite NaFePO4 as a high-performance electrode for Na-ion batteries,” *Energy Environ. Sci.*, vol. 8, no. 2, pp. 540–545, 2015, doi: 10.1039/c4ee03215b.

[5] C. Li, X. Miao, W. Chu, P. Wu, and D. G. Tong, “Hollow amorphous NaFePO4 nanospheres as a high-capacity and high-rate cathode for sodium-ion batteries,” *J. Mater. Chem. A*, vol. 3, no. 16, pp. 8265–8271, 2015, doi: 10.1039/c5ta01191d.

[6] P. P. Prosini, C. Cento, A. Masci, and M. Carewska, “Sodium extraction from sodium iron phosphate with a Maricite structure,” *Solid State Ionics*, vol. 263, pp. 1–8, 2014, doi: 10.1016/j.ssi.2014.04.019.

[7] M. Chen *et al.*, “NASICON-type air-stable and all-climate cathode for sodium-ion batteries with low cost and high-power density,” *Nat. Commun.*, vol. 10, no. 1, 2019, doi: 10.1038/s41467-019-09170-5.

[8] X. Zhu *et al.*, “A new sodium iron phosphate as a stable high-rate cathode material for sodium ion batteries,” *Nano Res.*, vol. 11, no. 12, pp. 6197–6205, 2018, doi: 10.1007/s12274-018-2139-0.

[9] P. Singh, K. Shiva, H. Celio, and J. B. Goodenough, “Eldfellite, NaFe(SO4)2: an intercalation cathode host for low-cost Na-ion batteries,” *Energy Environ. Sci.*, vol. 8, no. 10, pp. 3000–3005, 2015, doi: 10.1039/c5ee02274f.

[10] H. Gao, I. D. Seymour, S. Xin, L. Xue, G. Henkelman, and J. B. Goodenough, “Na 3 MnZr(PO 4 ) 3 : A High-Voltage Cathode for Sodium Batteries,” *J. Am. Chem. Soc.*, vol. 140, no. 51, pp. 18192–18199, 2018, doi: 10.1021/jacs.8b11388.

[11] Y. Zhao, X. Gao, H. Gao, H. Jin, and J. B. Goodenough, “Three Electron Reversible Redox Reaction in Sodium Vanadium Chromium Phosphate as a High-Energy-Density Cathode for Sodium-Ion Batteries,” *Adv. Funct. Mater.*, vol. 30, no. 10, 2020, doi: 10.1002/adfm.201908680.

[12] M. Law, V. Ramar, and P. Balaya, “Na2MnSiO4 as an attractive high capacity cathode material for sodium-ion battery,” *J. Power Sources*, vol. 359, pp. 277–284, 2017, doi: 10.1016/j.jpowsour.2017.05.069.

[13] H. Li *et al.*, “Highly efficient, fast and reversible multi-electron reaction of Na_3_MnTi(PO4)_3_ cathode for sodium-ion batteries,” *Energy Storage Mater.*, vol. 26, pp. 325–333, 2020, doi: 10.1016/j.ensm.2019.11.004.
